# Doppler-Guided Second-Look Endoscopy in Peptic Ulcer Bleeding: A Randomised Controlled Trial

**DOI:** 10.3390/jcm12216722

**Published:** 2023-10-24

**Authors:** Michael Milek Nielsen, Ove B Schaffalitzky de Muckadell, Stig Borbjerg Laursen

**Affiliations:** 1 Department of Gastroenterology, Odense University Hospital, Sdr. Boulevard 29, 5000 Odense, Denmark; michael.milek.nielsen2@rsyd.dk (M.M.N.);; 2 Department of Clinical Research, University of Southern Denmark, J.B. Winsløws Vej 19, 5000 Odense, Denmark

**Keywords:** peptic ulcer haemorrhage [MeSH], doppler endoscopic probes, rebleeding

## Abstract

Background: Endoscopic treatment guided by Doppler endoscopic probes (DEPs) during index endoscopy may be associated with improved outcome in patients with peptic ulcer bleeding (PUB). As competencies for DEP evaluation are not always available for index endoscopy, we examined the outcome associated with DEP evaluation at second-look endoscopy. Methods: The study was designed as a non-blinded, parallel group, randomised controlled trial. Patients admitted with PUB from Forrest Ia-IIb ulcers, controlled by endoscopic therapy, were randomised (1:1 ratio) to second-look endoscopy <24 h with DEP evaluation of the bleeding ulcer or continued standard treatment. Patients were followed up for 30 days. The primary outcome was rebleeding. Secondary outcomes included the number of transfusions, length of hospital stay, and 30-day mortality. Results: A total of 62 patients were included. At second-look endoscopy, 91% (29/32) of patients had a positive DEP signal at the ulcer base and were treated with contact thermal therapy (n = 29), injection of diluted adrenaline (n = 23), and haemoclips (n = 7). Among the 32 patients treated with DEP evaluation, only one rebled (3%) compared to four patients (13%) in the control group (*p* = 0.20). There were no differences in the secondary outcomes between groups, and there were no complications related to DEP evaluation. Conclusions: Second-look endoscopy with DEP-guided evaluation and treatment is safe and associated with a very low risk of rebleeding (3%) in patients with PUB. Second-look endoscopy with DEP evaluation may be considered in selected PUB patients at high risk of rebleeding, and may represent an alternative to the use of DEP for index endoscopy. Nevertheless, we did not find that second-look endoscopy with DEP evaluation significantly improved patient outcome compared to standard treatment.

## 1. Introduction

A recent study found that the incidence of peptic ulcer bleeding (PUB) increased from 40.1/100,000 in 2014 to 49.9/100,000 in 2019 in the United States [1]. Rebleeding occurs in 11–13% of patients, and is associated with a two-to-three-fold increase in mortality [2,3]. In order to decrease the rate of rebleeding, and thereby improve mortality rates, new and better treatments are needed to help achieve permanent haemostasis. 

International guidelines recommend the use of stigmata for recent haemorrhages (SRHs) at the ulcer base as a visual guide for the risk stratification and evaluation of the need for endoscopic intervention in PUB [4,5]. SRH is categorised according to the Forrest Classification (Supplementary Table S1) [6]. Endoscopic therapy is recommended for ulcers with active bleeding or visible non-bleeding vessels, whereas treatment for ulcers with an adherent clot is more debated due to lack of evidence [4,5]. One potential limitation of visually guided endoscopic therapy is that it in most cases is impossible to evaluate whether endoscopic treatment has led to permanent flow stop in the underlying vessel. 

During the past 25 years, several clinical studies and a meta-analyses have analysed the outcome associated with use of Doppler endoscopic probe (DEP) evaluation and Doppler-guided endoscopic therapy in patients with PUB [7,8,9,10,11,12,13,14]. Although there is some discrepancy between findings, previous studies indicate that a positive Doppler signal is an important predictor for risk of rebleeding [8,12,14], and that DEP-guided endoscopic therapy may improve patient outcome including reduced risk of rebleeding [7,11,13,14], surgery [7,11,14], and bleeding-related mortality [7,14].

One important drawback of DEP evaluation and treatment is the requirement of sufficient competencies of the attending endoscopist. In most medical centres world-wide, it will be hard to offer out-of-hours emergency endoscopy with DEP evaluation. This may, in part, be solved if similar positive results could be achieved by performing DEP evaluation and treatment at a repeat endoscopy performed within a short timeframe. 

The aim of this study was to evaluate whether DEP-guided evaluation and therapy, performed at a second-look endoscopy within 6–24 h from the index-endoscopy, could improve patient outcome in patients with PUB compared to standard treatment. 

## 2. Materials and Methods

### 2.1. Study Design and Randomisation

The study was designed as a single-centre, non-blinded, randomised controlled superiority trial of patients with PUB from ulcers with active bleeding, a visible non-bleeding vessel, or an adherent clot that could not be dislodged by forceful irrigation (Forrest I-IIb). Potential candidates received oral and written information about the study after the index endoscopy as soon as they were unaffected by sedation. Patients accepting to participate were randomised in a 1:1 ratio to 1. second-look endoscopy with DEP evaluation within 24 h from the index endoscopy (active arm) or 2. standard treatment (control group). Randomisation lists were constructed using a computer-generated sequence, and kept in consecutively numbered opaque envelopes. Participants were enrolled and assigned to their treatment by the primary investigator or the attending gastroenterologist.

The study was approved by the local research ethics committee (S-20130140) and followed the declaration of Helsinki. The study was registered at ClinicalTrails.gov (NCT02434978). Our report follows the CONSORT statement [15]. All the authors had access to the study data and have reviewed and approved the final manuscript.

### 2.2. Definitions

PUB was defined as presentation with hematemesis and/or melena, with subsequent upper endoscopy confirming the source to be a peptic ulceration. Rebleeding was defined according to the criteria by Laine and colleagues (Supplementary Table S2) [16].

### 2.3. Participants 

All patients presenting with PUB from Forrest I-IIb ulcers at Odense University Hospital, a thousand bed university hospital, during a period of 44 months were screened for inclusion. Patients with persistent bleeding at the time of index endoscopy, patients with life-expectancy of less than 30 days due to severe comorbidity, and patients with malignant disease in the upper GI tract were not considered for inclusion. Patients who developed PUB while an inpatient for another reason were considered for inclusion.

### 2.4. General Treatment

The general treatment of patients followed the Danish national guidelines for treatment of PUB [17] and the European Society of Gastrointestinal Endoscopy (ESGE) guidelines for management of nonvariceal upper gastrointestinal haemorrhage [4]. The index endoscopy was performed within 24 h of presentation to hospital. Endoscopic therapy was performed using injection of diluted adrenaline (1:10,000), heater probes, and/or haemoclips. Injection of diluted adrenaline was always combined with contact coagulation or haemoclips. Following endoscopic haemostasis, patients were treated with a 72 h infusion of esomeprazole (80 mg bolus followed by 8 mg/h). Patients were closely monitored at a specialised gastrointestinal bleeding unit for a minimum of three days.

### 2.5. Endoscopic Doppler Evaluation

Second-look endoscopy was performed by the primary investigator (SBL) in the period 6–24 h after the index endoscopy. The ulcer was examined with continuous wave ultrasound using the EZ-Dop^®^ from DWL (Cameron Park, CA, USA) with a 1.8 mm 16 Mhz endoscopic Doppler probe. Settings were as follows: scan depth 1 mm, SV 0.9, intensity spatial peak temporal average (ISPTA) 60, gain 80%, filter 160, and scale 10,000.

In order to ensure uniform DEP evaluation, all ulcers were evaluated using the same approach. First, the ulcer base was identified and cleansed using jet irrigation and suctioning, with removal of any clots or blood from the ulcer base. Then, the ultrasound probe was passed through the working channel of the endoscope and pressed firmly against the ulcer base. The ulcer base was scanned thoroughly for a minimum of five minutes for Doppler measurement. During DEP evaluation, the angle of the DEP was alternated between 0 and 180 degrees while ensuring optimal contact with the ulcer base. A positive DEP signal was defined as a repetitive, uniform spike-wave signal of a least three cycles, indicating a pulsatile flow. 

When obtaining a positive signal, the direction of the underlying vessel was further investigated using DEP. The area of the ulcer base with a positive Doppler signal was treated with a 10F heater probe until the Doppler signal disappeared. Ulcers with a continuous positive Doppler signal, despite heater probe treatment, were treated with injection of diluted adrenaline (1:10,000) or haemoclips until flow stop. 

### 2.6. Primary and Secondary Outcomes

The primary endpoint was rebleeding (see detailed definition in Supplementary Table S2) within five days of the index endoscopy. Secondary endpoints included 30-day mortality, length of hospital stay and number of blood transfusions received. Patients were followed up for 30 days.

### 2.7. Statistical Analyses

Proportions were compared using the Fischer’s exact test. Analyses of differences in continuous variables were performed using unpaired two-sample *t*-test. The appropriateness of underlying assumptions was examined prior to analysis. The Mann–Whitney U test was used for comparison of nonparametric data.

### 2.8. Sample Size Estimation

The required sample size was calculated based on the primary endpoint. We assumed that performance of second-look endoscopy with Doppler-guided evaluation and treatment could reduce the rate of rebleeding from 15% to 2% [7]. Using a power (1 − β) of 80% and a significance level (α) of 5%, inclusion of at least 86 patients in each arm was needed.

## 3. Results

### 3.1. Included Patients

**Figure 1:**
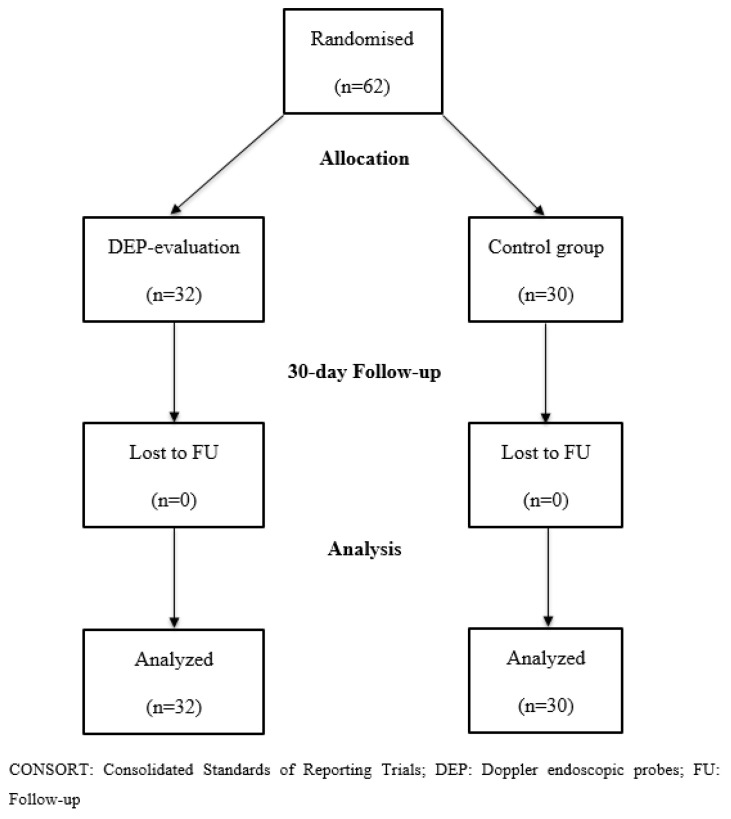
Consort flow diagram showing enrolment and analysis.

A total of 62 patients were included in the study (Figure 1). The mean age of patients was 72 years and 60% were males. The most common symptom at presentation was melena (92%). A total of 27% of patients were diagnosed with ischemic heart disease and 48% were treated with aspirin at time of hospital admission. The majority of patients bled from duodenal ulcers (38/62; 61%) and a visible non-bleeding vessel at the ulcer base was the most common stigmata of bleeding at index endoscopy (26/62; 42%). Five patients (8.1%) rebled within five days, and three patients (4.8%) died within 30 days. Further data on patients’ characteristics, symptoms at presentation, medication use, and findings at index endoscopy are summarised in Table 1. 

### 3.2. Doppler-Guided Evaluation and Treatment

Thirty-two patients were randomised to second-look endoscopy with Doppler evaluation. Among these patients, 29/32 (91%) had a positive DEP signal at second-look endoscopy and were treated with contact coagulation probes leading to flow stop in 6/29 (21%) of patients. Due to continuous Doppler flow despite contact thermal therapy—or an estimated need for further endoscopic treatment based on endoscopic evaluation (e.g., large protruding visible vessel)—23 (72%) patients were treated with an injection of diluted adrenaline and seven (22%) patients were treated with haemoclips. The median [95% CI] volume of injected diluted adrenaline was 14 mL [5–29]. There were no complications related to Doppler-guided evaluation or endotherapy.

### 3.3. Patient Outcomes

Rebleeding rates were 1/32 (3%) and 4/30 (13%) (*p* = 0.20) in the Doppler group and control group, respectively. The patient who rebled in the Doppler group had three ulcers in the duodenal bulb with diameters ranging from 5 to 15 mm. When performing DEP evaluation, one minor ulcer (5 mm) had a central protruding vessel with minor oozing. DEP evaluation identified underlying pulsating flow. The ulcer was initially treated with contact coagulation without achieving haemostasis. Following the injection of 9 mL of diluted adrenaline, the vessel was treated with a haemoclip. Despite haemostasis, repeated DEP measurement confirmed ongoing flow. Additionally 8 mL of diluted adrenaline was injected into the ulcer base. A third DEP measurement was not performed. Five days later, the patient developed rebleeding with haemodynamic instability and was treated with transarterial embolisation (TAE) of the gastroduodenal artery using coils. Three days later, the patient rebled again. This led to further treatment, including endoscopic therapy followed by repeated TAE of the superior and inferior branches of the pancreaticoduodenal artery, resulting in long-term haemostasis. Four patients in the control group developed rebleeding. In three of these patients, haemostasis could be obtained through repeat endoscopy with additional endoscopic therapy. The last patient was treated with surgery, preceded by a repeat endoscopy showing pulsating bleeding and signs of ulcer perforation.

Two patients (6%) died within 30 days in the Doppler group compared with one patient (3%; *p* = 1.00) in the control group. Regarding the causes of death, two patients died of respiratory failure and one was found dead in his house seven days after endoscopic treatment with no signs of bleeding. No autopsy was conducted. There were no bleeding-related deaths.

There were no differences in the length of hospital stay or number of transfusions received between groups (Table 2). As mentioned above, one patient in the control group was treated with surgery for severe rebleeding and suspected ulcer perforation that may have been caused/worsened by applied contact coagulation during index endoscopy. Aside from this, we have no knowledge of any important causes of harm or unintended effects among the study participants.

## 4. Discussion

Despite that several studies have indicated a positive outcome associated with DEP-guided evaluation and treatment, the use of DEP has never been implemented as a routine tool in everyday clinical practice. Although this may be explained by a relatively low level of evidence, we believe that implementation is limited by the requirement of the attending endoscopist to have the qualifications needed to perform DEP treatment during the index endoscopy. In many medical centres world-wide, it will be impossible to provide high-quality DEP evaluation out-of-hours. The idea of the present study was to clarify whether the use of DEP evaluation during second-look endoscopy is associated with improved patient outcome. The implementation of second-look DEP treatment only requires the DEP skills of a few endoscopists at each hospital, and is thus probably more realistic to implement in clinical practice in most medical centres. To the authors’ knowledge, we are the first to evaluate the use of second-look DEP treatment, and our results indicate that this strategy is safe and associated with a very low rate of rebleeding (3%). We were unable to show any statistical significant differences in patient outcomes when comparing second-look DEP treatment with standard treatment, which may be explained by the lack of power of the study.

Previous studies evaluating DEP-guided treatment during index endoscopy found rebleeding rates ranging from 2 to 20% [7,11,13]. This wide span in rebleeding rates reflects very different approaches in relation to Doppler measurement, type of endoscopic treatment (e.g., monotherapy with injection of diluted adrenaline [7]), and, in particular, patient selection (e.g., the inclusion of sources of bleeding other than PUB [13], the inclusion of patients with severe bleeding only [13], and the inclusion of ulcers with low-risk stigmata of bleeding [7]). These major differences make direct comparisons of study findings difficult. Overall, our findings indicate that the outcome associated with second-look endoscopy with DEP evaluation seems as good as the outcome associated with DEP-guided treatment during index endoscopy.

In order to secure DEP experience, DEP evaluation was only performed by one endoscopist (SBL). Furthermore, we tried to increase the internal validity by using a clear definition of a positive Doppler signal. It is noteworthy that a positive Doppler signal was found in 91% of patients, almost matching the 87.4% flow detection rate prior to endoscopic treatment in Forrest Ia, IIa, and IIb ulcers reported by Jensen and colleagues [12]. The high rate of positive Doppler signals may indicate problems with false-positive DEP measurements in the present study. In the study on DEP as a guide to risk stratification by Jensen et al. [12], residual blood flow was detected in 27.4% of patients with Forrest Ia, IIa, and IIb ulcers when performing DEP evaluation immediately after endoscopic haemostasis. A previous study using the injection of diluted adrenaline reported that 28% of patients who were Doppler-negative after endoscopic treatment during index endoscopy turned Doppler-positive at a repeat endoscopy the next day [10]. Therefore, based on previous studies, we did expect to find a relatively high rate of Doppler-positive patients.

To the authors’ knowledge, there are no data describing the learning curve for the DEP evaluation of patients with PUB. It is our experience that the performance of around 25 procedures is sufficient for an experienced endoscopist to be able to perform DEP evaluation. Although the implementation of DEP evaluation is associated with extra costs, a cost-effective analysis from the US found that the use of DEP evaluation (during index endoscopy) was cost-effective [18].

The main strength of the present study lies in the randomised controlled design and inclusion of a homogenous group of patients with peptic ulcer bleeding. A clear limitation is that the number of included patients was considerable lower than needed according to our sample size calculation. The study was originally designed as a multicentre study with the inclusion of patients from three high-volume medical centres in Denmark. Unfortunately, patient inclusion at two medical centres was too insufficient to include in the study. A post hoc analysis showed a power of 31% for the outcome of rebleeding, leading to a high risk of performing a type 2 error. However, we are not concluding that DEP evaluation is associated with a similar outcome to standard treatment. We present a new treatment modality in patients with PUB that, based on our observations, seems to be interesting and associated with a good outcome (3% rebleeding rate and no procedure-related complications). The use of blinding of the attending physicians would have increased the quality of the study, but as the main outcomes of interest (rebleeding and mortality) were clearly defined, we do not believe that the lack of blinding had any significant impact on the main conclusions of the study.

## 5. Conclusions

In conclusion, the results of the present study indicate that second-look endoscopy with DEP-guided evaluation and treatment is a safe method associated with a very low risk of rebleeding (3%) in patients with PUB. Second-look endoscopy with DEP evaluation may represent an alternative to the use of DEP during index endoscopy. Future studies are needed in order to examine whether second-look endoscopy with DEP evaluation is associated with a similar patient outcome to the use of DEP during index endoscopy. Additionally, further studies are essential to clarify with certainty whether or not the use of DEP is associated with improved patient outcome when compared with traditional endoscopic treatment.

## Figures and Tables

**Table 1:** Patient characteristics.

	DEP Group (n = 32)	Control Group (n = 30)	Total (n = 62)
General characteristics			
- Age (years, median [95% CI])	72 [53–90]	73 [53–90]	72 [53–90]
- Sex; male	22 (69)	15 (50)	37 (60)
Symptoms at presentation			
- Haematemesis	13 (41)	11 (37)	24 (39)
- Melaena	29 (91)	28 (93)	57 (92)
- Haematochezia	3 (9.4)	1 (3.3)	4 (6.5)
- Syncope	6 (19)	6 (20)	12 (19)
Comorbidities			
- Cancer	3 (9.4)	5 (17)	8 (13)
- COLD	6 (19)	5 (17)	11 (18)
- Diabetes	6 (19)	4 (13)	10 (16)
- Ischaemic heart disease	11 (34)	6 (20)	17 (27)
- Liver cirrhosis	1 (3.1)	1 (3.3)	2 (3.2)
- Renal disease	7 (22)	2 (6.7)	9 (15)
- ASA-score (mean, [95% CI])	2.7 [2–3]	2.4 [1–3]	2.6 [2–3]
Medication			
- ADP-RI	6 (18.8)	2 (6.7)	8 (13)
- Aspirin	17 (53)	13 (43)	30 (48)
- Direct thrombin inhibitor	0 (0)	1 (3.3)	1 (1.6)
- Factor Xa inhibitor	2 (6.3)	1 (3.3)	3 (4.8)
- NSAIDs	12 (38)	7 (23)	19 (31)
- Unfractionated heparin	2 (6.3)	3 (10)	5 (8.1)
- Vitamin K antagonist	3 (9.4)	2 (6.7)	5 (8.1)
Haemodynamic parameters at presentation			
- Systolic blood pressure (mmHg, mean [95% CI])	113 [74–157]	123 [90–153]	118 [80–154]
- Heart rate (beats/min, mean [95% CI])	94 [60–122]	97 [56–166]	96 [60–126]
Blood tests			
- Haemoglobin (g/dL, mean [95% CI])	5.1 [3.0–7.3]	5.2 [3.2–7.4]	5.2 [3.2–7.4]
- Albumin (g/L, mean [95% CI])	31 [21–40]	32 [23–42]	31 [22–40]
- Urea (mmol/L, mean [95% CI])	20 [6.5–41]	18 [7.4–30]	19 [7.1–35]
- Creatinine (µmol/L, mean [95% CI])	139 [46–360]	100 [53–160]	120 [53–246]
Ulcer characteristics			
- Duodenal ulcer location	22 (69)	16 (53)	38 (61)
- Stigmata of bleeding (Forrest)			
- 1a	2 (6.3)	2 (6.7)	4 (6.5)
- 1b	12 (38)	10 (33)	22 (35)
- 2a	11 (34)	15 (50)	26 (42)
- 2b	7 (22)	3 (10)	10 (16)

ADP-RI: adenosine diphosphate receptor inhibitor; ASA: American Society of Anaesthesiologists score; CI: confidence interval; COLD: chronic obstructive lung disease; DEP: Doppler endoscopic probes; NSAIDs: nonsteroidal anti-inflammatory drugs. Data are n (%), unless otherwise stated. There were no missing values for reported variables.

**Table 2:** Patient outcomes.

	DEP Group (n = 32)	Control Group (n = 30)	Total (n = 62)	*p*
Length of hospital stay (days, median [95% CI])	4 [3–20]	4 [3–30]	4 [3–20]	0.72
No. of blood transfusions (median [95% CI])	0 [0–2]	0 [0–3]	0 [0–3]	0.96
Rebleeding (n(%))	1 (3.1)	4 (13)	5 (8.1)	0.20
30-day mortality (n(%))	2 (6.3)	1 (3.3)	3 (4.8)	1.00

CI: confidence interval; DEP: Doppler endoscopic probes. There were no missing values for reported variables.

## Data Availability

The data presented in this study are available on request from the corresponding author.

## Supplementary Materials

The following supporting information can be downloaded at: https://www.mdpi.com/article/10.3390/jcm12216722/s1, Table S1: Forrest Classification; Table S2: Criteria for rebleeding *.
