# Supplementary material for: Doppler-Guided Second-Look Endoscopy in Peptic Ulcer Bleeding: A Randomised Controlled Trial
Source: J Clin Med. 2023 Oct 24;12(21):6722. doi: 10.3390/jcm12216722 (PMC10647717; doi:10.3390/jcm12216722)
Supplement: Supplementary file 1 [file jcm-12-06722-s001.zip › jcm-2578246-supplementary.pdf]

Supplementary Table S1. Forrest Classification

Forrest Classification	Stigmata of bleeding at ulcer base
Ia	Spurting bleeding
Ib	Oozing bleeding
IIa	Non-bleeding visible vessel
IIb	Adherent clot
IIc	Hematin on ulcer base
III	Clean base ulcer

Adapted from Forrest et al.[6]

Supplementary Table S2. Criteria for rebleeding *.

Hematemesis or bloody nasogastric aspirate > 6 hours after endoscopy
Melena after normalization of stool color
Hematochezia after normalization of stool color or after melena
Development of tachycardia (heart rate ≥110 beats per minute) or hypotension (systolic blood pressure ≤ 90mmHg) after at least one hour of hemodynamic stability in the absence of an alternative explanation for hemodynamic instability such as sepsis, cardiogenic shock, or medication.
Hemoglobin drop of ≥2g/dL after two consecutive stable hemoglobin values (<0.5g/dL decrease) ≥ 3 hours apart
Tachycardia or hypotension that does not resolve within 8 hours after index endoscopy despite appropriate resuscitation (in the absence of an alternative explanation), associated with persistent melena or hematochezia
Persistently dropping hemoglobin of > 3 g/dL in 24 hours associated with persistent melena or hematochezia

* Rebleeding was defined as fulfillment of one of these criteria. Adapted from Laine et al.[16]
